# Hôtel Dieu

**Published:** 1829-07-01

**Authors:** 


### CLINICAL REVIEW.

### LI.

### HOTEL DIEU.

### Treatment of Imperforate Rectum.*

Imperforate rectum, or imperforate anus, is not only a dangerous, but unhappily a much too frequent malformation, and one in which surgery alone can be of service. At page 515 of the last number of this Journal, we related the particulars of an interesting case which occurred in Paris to M. Piédagnel, and we shall now advert briefly to one or two others that have since been recorded.

In February of the present year, an infant, three days old, was brought to M. Dupuytren at the Hôtel Dieu, with the anus well formed, but opening into a cul-de-sac, which admitted the extremity of the little finger. The cul-de-sac appeared to be in contact at its apex with the rectum dilated by meconium, and pressure on the hypogastrium rendered this sensation communicated to the finger still more distinct. The accoucheur had made some unsuccessful attempts to open the intestine with a lancet. The belly was tense, tympanitic, and painful—there was frequent vomiting—and, under these alarming circumstances, M. Dupuytren was afraid to adopt the method of Durat, and establish an artificial anus in the left groin. Instead of this procedure M. D. introduced a trocar in the natural direction of the rectum, but only gave issue to a little gas, and the poor infant died in the course of the day.

At the same time that this case occurred, another child, five days old, was brought for the inspection of the same distinguished surgeon. In this there was no cul-de-sac, and the anus was only slightly marked—there had been some vomiting—belly slightly tense and painful. In this, as in the former instance, a lancet had been introduced without success. M. Dupuytren wished to open into the descending colon in the left groin, but the parents refused their consent. A puncture was made with a trocar in the usual situation of the rectum, but nothing was discharged, and the infant was consigned to those who had the care of it.

The foregoing are melancholy instances of the fatal effects of imperforate rectum, and we turn with pleasure to one of a more pleasing cast, recorded by Mr. Miller in the January number of the Edinburgh Journal. We make no apology for noticing it shortly in this place, as it gives an additional interest to the subject.

In 1821, Mr. Miller attended Mrs. —

* Journ. Hebdomadaire, No. 23.

in her confinement, and assisted to bring into the world a male infant, who at first was supposed to be perfectly formed, but was soon discovered to want the natural outlet of the intestines. No trace of an anus existed, but the meconium was passed by the urethra, shewing that the rectum opened into the bladder. Mr. Miller made an incision with a scalpel in the situation of the anus, an inch and upwards in length and very deep. He then plunged a trocar in the supposed direction of the gut, and fortunately for the child, and to its great relief, meconium issued through the canula at the second attempt. When the canula was removed, pieces of sponge-tent were introduced, but they could not be borne for any time, and injections of warm gruel were employed. In spite of every care, however, the aperture narrowed so much during cicatrization, that it was necessary to enlarge it ten times by a bistoury in the course of eight months. The child grew, but with its growth contracted an unfortunate penchant for coal-cinders, which he constantly eat, and which almost as constantly stuck in the passage, and required frequent operations to remove them. In one of these the bladder was wounded, and an urinary fistula in the rectum was the consequence. He possessed the power over the sphincter ani, but required the frequent use of laxatives and glysters; in administering which about three years ago the mother perceived that some substance opposed the introduction of the pipe. Little, however, was said about it, till at length the obstruction of the bowels was complete, and Mr. Miller on examination found a calculous concretion of very large size, blocking up the passage, and felt with a probe through the orifice of the anus.

"As the anus would only admit a goose-quill, I enlarged it sufficiently to allow of a full examination with my finger, when, finding the stone almost to fill the hollow of the sacrum, it was useless to think of extracting it entire, as I felt satisfied that the bones alone would absolutely frustrate every such attempt. Drilling it to pieces by boring in different directions, at once struck me as the only method of proceeding, and I unwillingly left the little sufferer for another day, that I might provide the appropriate instruments. Having procured, from an ingenious mechanic, a drilling apparatus to be wrought with a bow and string, (resembling a common hand-drill,) I had a few drill-bits formed of a conical shape and of various sizes, to make a bore of from $\frac{1}{4}$ to $\frac{5}{8}$ths of an inch in diameter. The greatest difficulty appeared to be the properly fixing the stone when in the act of drilling. The impossibility of introducing the usual forceps to grasp such a stone, suggested to me the propriety of having a pair constructed on purpose with separate blades, which might be fixed with a screw when applied; and such a pair being promptly prepared by Mr. Marshall, cutler, and having obtained the valuable assistance of my friend, Mr. James Miller, surgeon at Perth, I enlarged the anus in the direction of the sacrum as far as the coccyx would allow, and in the direction of the pubes, as far as the safety of the urethra would permit. The separate blades of the forceps were now cautiously introduced, and secured by turning the screw. I then applied the drilling apparatus, while my friend, Mr. Miller, held the stone firmly at the orifice betwixt the blades of the forceps, using every precaution to avoid injury. The drills were steadily and perseveringly applied, first of the small and then of the larger size, until considerable openings were made in the stone in various directions. A pair of strong polypus forceps was now thrust into the openings, and the blades being forcibly separated by extending the handles, the calculus gave way, and was separated into three pieces, each of which had in its turn to be drilled and broken in the same manner before it could be extracted. The whole operation occupied not less than two hours and three quarters; and it was truly surprising to see with how much fortitude the little fellow bore such protracted suffering."

No bad symptoms of any kind followed. The patient had perfect power over the sphincter ani—and Mr. Miller believes that, under the use of the elastic gum catheter, the vesico-rectal fistula will heal. The calculus was at least the size of a turkey's egg, but the *practical* portion of the case being what we want, we need not stop to describe its chemical composition. The proceeding adopted does much credit to Mr. Miller's ingenuity, and may serve as a precedent in a similar case. It is obvious that the *lithontripteur* of M. Civiale, or still more the improvement upon it carried into effect by Dr. Pecchioli, of Florence, and described in a recent number of this Journal, would be infinitely preferable to a comparatively clumsy and unhandy complication of instruments.

II. Treatment of the Ranula by Seton.

It seems that a Dr. Langier has recently *invented* this plan of treatment in Paris, and entered into some learned disquisitions to prove that it may succeed. The Doctor, however, might have saved himself the trouble of discussing the matter at any great length, as the identical proceeding has long been adopted at St. George's Hospital by Mr. Brodie, and we dare to say by others elsewhere. It was but in the 20th number of our Journal, that we gave an account of cases of ranula treated with more or less success in this manner.

III. Supposed Hernia of the Lung, in consequence of Tight Stays.*

A young woman, 18 years of age, of good constitution, and who had menstruated regularly, presented herself to M. Breschet with a tumour in the right side of the neck. It rose from behind the clavicle to as high as the level of the thyroid cartilage; was broader at its base than at its apex; was of variable size, but at its maximum about the dimensions of the fist; on compression, felt elastic and soft; disappeared entirely if the pressure was made from above downwards and inwards, but again returned on withdrawing the pressure, especially if the stays were laced at the time; was perfectly indolent, and presented no pulsation. On applying the stethoscope to the tumour, the respiratory murmur was distinctly heard, and no doubt remained, from this and the preceding symptoms, that the enlargement was produced by the summit of the lung protruded from the cavity of the chest; in other words, by a hernia of that organ. The disease was produced by the habit in which the young woman, like most other females of her age, had indulged, of wearing the stays exceedingly tight laced. When she did this she always experienced a difficulty of respiration, and the volume of the tumour increased. M. Breschet directed this patient to leave off her stays, and proposed to apply compression to the surface of the hernia.

* Clinique, No. 5, Tome 4.

IV. Hernia, Absence of the Testicles from the Scrotum.

On the 3d of February, M. Sanson exhibited to the éléves of the Hôtel Dieu, a man 35 years of age, who had entered the hospital for strangulated inguinal hernia. The gut had been returned by the taxis, but the point of curiosity was the absence of both the testicles from the scrotum. The left was felt in the inguinal canal, but the right could not be discovered at all. From what we have seen we should say, that the imperfect descent of the testicle is a much more frequent occurrence than is generally imagined, or, indeed, than we are told in books. If the testicle thus situated in the inguinal passage should chance to inflame, the case not only may be, but has been mistaken for hernia, and treated accordingly. The fasti of surgery have more than one error of this kind on their pages. When a real and bonâ fide hernia is combined with the mal-position of the testis, the case is also perplexing, especially if an operation is required to be performed. An interesting case of this description lately occurred to Mr. Hawkins at St. George's Hospital; the operation was performed, and the patient did well.

V. Popliteal Aneurism—Operation—Gangrene.*

Car—— æt. 35, clerk to the barriers,

* Clinique, Tome 3, No. 96.

who had lived hard, and been subject to extreme privations, began, at the age of 32, to be affected with dyspnœa and palpitations of the heart. During a march of more than 500 leagues he was seized with pain in the right ham, and when he had arrived in France, he discovered a violent pulsation in this part, accompanied with lancinating pains, and augmented by walking or exertion. This was in September, and by the 1st of last January he was quite laid up by his complaint, for which he entered the Hôtel Dieu on the 16th of the next month. He was pale, flabby, and emaciated; affected with head-ache and vertigo; the action of the heart was violent; and all the arteries accessible to the touch, presented to the sight the same unnatural pulsation. The tumour itself in the ham had all the usual characters of aneurism; was very painful in the direction of the peroneal division of the sciatic; and the limb below was œdematous. The health was good in all other respects.

After bleeding from the arm and opening the bowels by castor oil, the operation of tying the femoral artery in the upper third of the thigh was performed by M. Dupuytren. On arriving at the sheath it was found to be thickened, indurated, and apparently the seat of chronic inflammation. The artery was secured with a single thread, on tying which the pulsations in the tumour, as well as in the limb, were suspended. Soon after the operation the patient was seized with excruciating pain on the outer side of the leg, stretching towards the foot and ankles; the face became flushed; and the pulse got up to 140. He was bled to three *palettes* and took an anodyne, which procured him some sleep during the night, but on the 21st the pain was still severe, the temperature of the limb was rather low, and some feeble pulsations were felt in the knee, and even on the dorsum pedis. Next day there was much depression, pulse 135, countenance pale, bowels not open. In the course of the day he had epistaxis, and in the evening was bled. During the night he had two short fits of syncope. On the 22d, the whole surface of the limb was warm, but on the upper and outer side of the thigh was a patch of erysipelatous redness, with a doughy state of the subcutaneous cellular membrane. The pulse was 118—urine scanty and high-coloured—irritability excessive—aneurismal tumour diminished in size—the wound looking well. A poultice to the thigh, diluents, and Seltzer water, were the means employed, and on the 24th he was generally better, though the lower and external part of the calf was still much swollen and painful.

On the 25th, the premonitory appearances of gangrene disclosed themselves in the alteration of the countenance, diminished sensibility and temperature, and slight œdematous swelling of the foot. The limb was kept warm and raised, and broths, vin sucré, and anodynes administered. He passed a bad night, and the next day only shewed the gangrene more developed in the foot and inferior part of the leg. From the 26th to the 2d of March, the destruction of the parts went slowly on, the foot passed into complete mortification, large phlyctenæ covered the leg, the thigh at its upper and outer part swelled, and furnished, on incision, a great quantity of pus, and the opposite limb became œdematous. In the midst of all this, the system stood its ground in a remarkable degree. On the 9th, the ligature came away from the femoral artery, and was followed by no hæmorrhage; the wound was filled with pus, which burrowed in the thigh; but the intellectual faculties were still entire. From this till the 18th he gradually got lower, and, after sinking into a lethargic calm, expired at 11, p.m. of that day, the 26th after the operation. The mortification had not extended above the parts already mentioned, although no line of separation formed. The foot itself dried, and resembled that of a mummy, in fact, its condition was what has been denominated the "dry gangrene."

*Sectio Cadaveris.* The small intestines contained some living tape-worms, and the mucous membrane with which they were in contact was not in the least inflamed. The left side of the heart was hypertrophied to double its ordinary volume, the parietes of the ventricle being upwards of an inch in thickness. Its texture was bright red and firm, its lining membrane and valves unaltered. The aorta was very large, and presented the atheromatous deposits between its coats in abundance; it had no patches of ossification. The same atheromatous depositions were found in the descending trunk, and more or less in the whole arterial system. The carotids which, during life, had appeared to be greatly dilated, were shewn on dissection to be very little so. The sheath of the right femoral artery was dense and indurated. The ligature had been placed on the vessel three fingers' breadth below the rise of the profunda. The artery had not ulcerated at the spot where the ligature had been, but was sealed by a clot five lines in length above the ligature, and a little less below it. The aneurism in the ham was mainly formed by dilatation only of the coats; at its lower part, however, the inner had been destroyed, and presented a circular aperture leading to a pouch formed by the outer. In this, but not in the larger sac, were some fibrinous clots. The popliteal vein adhered strongly to the sac, and in this situation its cavity was entirely obliterated. The venous circulation was carried on by one of the veins on the calf of the leg having enlarged, and opened into the saphena minor. There were many collections of pus around the femoral artery and between the muscles on the outside of the thigh. In the leg was also a similar deposition. No inflammation of the veins in any part could be discovered.

The above case is of great practical interest and importance. It teaches the surgeon to beware of the operation for aneurism in that state of excessive excitement of the circulating system which is marked by preternatural pulsation in a most all the tangible arteries, and tumultuous action of the heart. In such a case the operation seldom or never succeeds, for the aneurismal diathesis, if we may use the expression, is so strongly rooted in the system, that secondary hæmorrhage, or some other ill consequence generally follows the application of the ligature. Of this we have seen more than one example. The indurated state of the sheath of the femoral artery observed in the present case is a circumstance which occasionally happens, and is to be considered, we suppose, as another unfavourable item in the catalogue of symptoms In a case of femoral aneurism for which the usual operation was performed, this condition of the sheath, and indeed if our memory serves us, of the artery also, existed to a great degree. The patient sank from suppuration in the sac, and around the vessel.

---

